# Prognosis of patients with implanted pacemakers in 4‑year follow-up: Impact of right ventricular pacing site

**DOI:** 10.1007/s00059-017-4561-6

**Published:** 2017-04-10

**Authors:** K. Krzemień-Wolska, A. Tomasik, E. Nowalany-Kozielska, W. Jacheć

**Affiliations:** 1 Szpital Specjalistyczny w Zabrzu, Zabrzu, Poland; 2 Second Department of Cardiology, Medical University of Silesia, ul. Skłodowskiej 10, 41-800 Zabrzu, Poland

**Keywords:** Arrhythmias, Right ventricle, Mortality, Heart conduction system, Cardiac pacing, artificial, Arrhythmien, Rechter Ventrikel, Mortalität, Kardiales Erregungsleitungssystem, Herzschrittmacherstimulation

## Abstract

**Background:**

Pacing remains the method of choice for treatment of cardiac electrical conduction disorders.

This study examined the interrelationship between the site of the right ventricular lead tip and patient prognosis in association with other cardiovascular risk factors over a 4‑year follow-up period.

**Patients and methods:**

The study comprised 450 consecutive patients (223 women; aged 69.16 ± 9.63 years) who had their first SSI or DDD pacemaker implanted for typical indications.

**Results:**

During follow-up, 91 (20.2%) patients died. The positive prognostic factors were: female sex (hazard ratio [HR] = 0.426), DDD pacemaker (HR = 0.526), oral anticoagulant use (HR = 0.330; all groups), sodium concentration (HR = 0.926), oral anticoagulant (HR = 0.115) and statin (HR = 0.260) use (female group), and non-apical location of the right ventricular lead tip (HR = 0.549; male group). Risk factors for death were: age (HR = 1.063), diabetes requiring insulin (HR = 2.832), creatinine concentration (HR = 1.005; all groups), age (HR = 1.11; female group), and elevated creatinine level (HR = 1.012; male group). In all patients, the non-apical location of the right ventricular lead tip was associated with an 18.92% reduced mortality rate during the 4‑year follow-up, which was statistically significant for the male group.

**Conclusion:**

The non-apical location of the right ventricular lead tip was a positive prognostic factor and was statistically significant in the male subgroup.

## Zusammenfassung

**Hintergrund:**

Ein Schrittmacher bleibt die Methode der Wahl für die Behandlung kardialer elektrischer Erregungsleitungsstörungen. In der vorliegenden Studie wurde die Beziehung zwischen der Lage der rechtsventrikulären Elektrodenspitze und der Prognose des Patienten in Zusammenschau mit anderen kardiovaskulären Risikofaktoren im Verlauf einer 4‑jährigen Nachbeobachtungsphase untersucht.

**Patienten und Methoden:**

An der Studie nahmen 450 konsekutive Patienten teil (davon 223 Frauen; Alter: 69,16 ± 9,63 Jahre), bei denen erstmals ein SSI- oder DDD-Schrittmacher aus typischer Indikation implantiert wurde.

**Ergebnisse:**

Während der Nachbeobachtungsphase verstarben 91 (20,2%) Patienten. Die positiven prognostischen Faktoren bestanden aus: weiblichem Geschlecht (Hazard Ratio, HR: 0,426), DDD-Schrittmacher (HR = 0,526), orale Antikoagulanziengabe (HR = 0,330; alle Gruppen), Natriumkonzentration (HR = 0,926), Gabe von oralen Antikoagulanzien (HR = 0,115) und Statinen (HR = 0,260; Gruppe der Frauen) und nichtapikaler Lokalisierung der rechtsventikulären Elektrodenspitze (HR = 0,549; Gruppe der Männer). Risikofaktoren für Tod waren: Alter (HR = 1,063), insulinpflichtiger Diabetes (HR = 2,832), Kreatininkonzentration (HR = 1,005; alle Gruppen), Alter (HR = 1,11; Gruppe der Frauen) sowie erhöhter Kreatininspiegel (HR = 1,012; Gruppe der Männer). Bei allen Patienten war die nichtapikale Lage der rechtsventrikulären Elektrodenspitze mit einer 18,92% verminderten Mortalitätsrate während der 4‑jährigen Nachbeobachtungsphase assoziiert, was für die Gruppe der Männer statistisch signifikant war.

**Schlussfolgerung:**

Die nichtapikale Lage der Elektrodenspitze im rechten Ventrikel stellte einen positiven prognostischen Faktor dar und erwies sich in der männlichen Studienkohorte als statistisch signifikant.

For over 50 years, pacing has been the method of choice in the treatment of electrical conduction disorders [1–5]. The apical location for pacing was preferred since it provided the possibility to stabilize the location of the ventricular lead. The currently available active-fixation leads offer freedom of choice regarding the right ventricular (RV) pacing site. This is important because of the adverse consequences of long-term pacing of the RV apex [6, 7].

It was reported that apical pacing leads to impaired segmental shortening, reduction in the speed of pressure increase in the initial contraction period, abnormalities in contraction geometry, and hemodynamic disorders [8–10]. In addition, apical pacing extends the time of relaxation and shortens the time of filling of the left ventricle, which can cause ineffective contraction of the interventricular septum (as in the case of left bundle branch block) and leads to a decrease in stroke volume, impairment of mitral valve function, and an increase in telesystolic volume [11].

In addition to the aforementioned disorders, there are also adverse changes in coronary flow [12]. These changes have negative metabolic consequences for the heart muscle, which are evident in increased oxygen-free metabolism [13, 14] and decreased left ventricular ejection fraction (LVEF) [15].

Although the majority of studies confirm the adverse consequences of long-term pacing, especially of the RV apex [16–19], unequivocal indications for the optimal location of the right ventricular lead tip (RVLT) are still not defined.

Non-apical pacing of the right ventricle (RV) diminishes the influence of adverse pacing on the contraction desynchronization of the left ventricle and the associated poor electrical and hemodynamic cardiac function [20–24].

The RV outflow tract still remains the most frequently selected alternative site for ventricular lead implantation [25–27].

A meta-analysis of 14 studies confirmed the protective influence of non-apically stimulated RV with regard to echocardiographic and laboratory parameters as well as physical sufficiency and life quality [28]. However, data on the relation between the site of RV pacing and long-term prognosis are still scarce and ambiguous.

The objective of this study was to evaluate whether there is an interrelationship between the site of the RVLT and prognosis in patients with implanted pacemakers over a 4‑year observation period, taking other cardiovascular risk factors into consideration.

## Methods

The clinical data of subsequent patients who had single- or dual-chamber (VVI, DDD) pacemakers implanted for typical indications during the period 2006–2008 at the II Department of Cardiology of the Medical University of Silesia were retrospectively analyzed.

The observation period of the study was 4 years. The following inclusion criteria were applied: patients aged 18–80 years with typical indications for heart pacing (atrioventricular block, sick sinus syndrome, atrial fibrillation with slow ventricular action); LVEF greater than 40%.

The exclusion criteria were: patients with concurrent diseases significantly influencing the predicted lifespan (active cancer disease, severe kidney failure, persons abusing alcohol or narcotics, poorly controlled mental health disorders, acute coronary syndrome in the last 6 months). The study endpoint was all-cause mortality. Information on death was obtained from the National Health Fund database.

### Study parameters

The demographic data collected included information on the patients’ sex and their age at the time of implantation. The clinical data comprised indications for implantation and concurrent diseases (arterial hypertension, ischemic heart disease, diabetes, history of brain stroke).

Use of the following pharmaceuticals was also noted: beta-blockers (BB), angiotensin-converting enzyme inhibitors (ACE-I), angiotensin receptor blockers (ARB), thiazide and loop diuretics, statins, acetylsalicylic acid (ASS), oral anticoagulants (AVK), digitalis, oral anti-diabetic drugs, insulin therapy, and aldosterone antagonists (AA).

Further evaluations included laboratory parameters (hemoglobin, creatinine, cholesterol, and sodium concentrations) as well as echocardiographic parameters (left ventricular end-diastolic volume, LVEF).

Finally, data on the implantation procedure were also collected, i.e., the pacing mode (VVI/DDD), and the RVLT location. The decision concerning the location of the RVLT was left up to the operator.

### Statistical analysis

Constant variables with normal distribution are shown as means with standard deviation; the median value as well as lower and upper quartiles are presented. Qualitative parameters are presented as number of cases and percentages.

The comparative analysis of constant variables was conducted using the Student *t* test, whereas dichotomous variables were compared using the chi-square test with Yates’s correction.

A Cox regression model was used to define risk (hazard ratio) of death. Initial evaluation of the significance of potential prognostic factors was performed using the univariable Cox regression model. Factors with a prognostic value of *p* < 0.1 were included in the multivariable analysis. In the uni- and multivariable analyses, the risk of failure is presented as a hazard ratio (HR) with a 95% confidence interval (CI). The probability of event-free survival during the observation period for the selected variables was analyzed using the Kaplan–Meier method; statistically significant differences in the curves were evaluated using a log-rank test, including complete and censored data.

Data on the mortality rate are presented as raw data. Analysis using age as a layer (age-adjustment) was also conducted, but the results did not differ from the raw data. Statistical significance was set at *p* < 0.05. All statistical analyses were conducted with the STATISTICA 10.0 program.

The patients were divided into two groups for analysis: those who survived the 4‑year observation period and those who died during that time; the patients were further divided into female and male subgroups. Additionally, a comparison of the distribution of the selected variables was made between groups on the basis of the RVLT location.

Our study was a retrospective analysis, not a medical experiment, and therefore did not require the approval of the ethics committee.

## Results

Among 621 patients who had a pacemaker implanted for the first time in the period 2006–2008, 450 met the inclusion criteria (223 women) at the age of 69.16 ± 9.63 years. During the observation period, 91 patients died (20.2%), including 30 women.

Patients who survived the 4‑year follow-up, compared with those who died during the follow-up period, more often had dual-chamber pacemakers implanted, were younger in terms of statistical significance, and more often had arterial hypertension. In this group, more patients used statins, ASS, and AVK, while fewer received insulin therapy and loop diuretics. In a subgroup of patients who died, higher creatinine concentrations were observed. No differences in non-apical right ventricular lead location (n-ARVLL) were observed in the whole study group, whereas in the subgroup of patients with VVI pacemakers, the apical lead location prevailed.

As also seen in the main study group, women who survived when compared to women who died during the 4-year follow-up were younger initially, more often had a DDD pacemaker implanted, more often received statins, and less often required insulin therapy. In the group of women who died during the follow-up period, higher concentrations of creatinine and lower concentrations of hemoglobin were observed as compared with those who survived the observation period.

In the male group, patients who survived the 4‑year observation period were younger initially, had lower creatinine concentrations, and more often used beta-blockers, acetylsalicylic acid, and statins. They also more often had an n-ARVLL.

Detailed characteristics of the study group and both the female and male subgroups, along with survival rates and the results of uni- and multivariable analysis in Cox hazard regression, are presented in Tables 1, 2, and 3.

**Table 1:** Baseline patient data and survival analysis (uni- and multivariable Cox regression)

	Entire group			Univariable Cox regression analysis			Multivariable Cox regression analysis		
	Entire group	Survival	Death	*p*	HR	95% CI	*p*	HR	95% CI
No. of patients	450 (100%)	359 (79.78%)	91 (20.22%)	–	–	–	–	–	–
Female	223 (49.6%)	193 (53.8%)	30^*^ (33.0%)	0.001	0.463	0.30–0.72	0.000	0.426	0.27–0.67
Mean age at implantation	69.16 ± 9.63	68.14 ± 10.10	72.30^***^ ± 6.95	0.000	1.063	1.03–1.10	0.004	1.047	1.02–1.08
DDD	302 (67.11%)	256 (71.31%)	46^ ***^ (50.55%)	0.000	0.441	0.29–0.66	0.004	0.526	0.34–0.82
VVI	148 (32.89%)	103 (28.69%)	45^***^ (49.45%)	–	–	–	–	–	–
VVI-ARVLL	100 (22.22%)	66 (18.38%)	34^***^ (37.36%)	–	–	–	–	–	–
DDD-ARVLL	196 (43.56%)	166 (46.24%)	30^*^ (32.97%)	–	–	–	–	–	–
n-ARVLL (all)	154 (34.2%)	127 (35.4%)	27^ns^ (29.7%)	0.259	0.772	0.49–1.21	–	–	–
SSS	197 (43.78%)	161 (44.85%)	36^ns ^(39.56%)	0.356	0.821	0.54–1.25	–	–	–
A-V block	193 (42.89%)	148 (41.23%)	45^ns^ (49.45%)	0.149	1.354	0.90–2. 04	–	–	–
FA with bradycardia	60 (13.33%)	50 (13.93%)	10^ns ^(10.99%)	0.457	0.779	0.40–1.50	–	–	–
CAD	141 (31.33%)	115 (32.03%)	26^ns^ (28.57%)	0.455	0.841	0.53–1.32	–	–	–
HA	213 (47.33%)	179 (49.86%)	34^ns^ (37.36%)	0.029	0.623	0.41–0.95	0.291	0.771	0.48–1.25
DM-2	98 (21.78%)	74 (20.61%)	24^ns ^(26.37%)	0.214	1.344	0.84–2.14	–	–	–
Stroke	24 (5.33%)	17 (4.74%)	7^ns^ (7.69%)	0.299	1.504	0.70–3.25	–	–	–
Hemoglobin (g/dl)	13.27 ± 1.65	13.31 ± 1.53	13.12^ns^ ± 2.05	0.303	0.933	0.82–1.06	–	–	–
Creatinine (µmol/l)	96.26 ± 8.10	84.12 ± 23.78	110.53^ns^ ± 57.24	0.000	1.008	1.01–1.01	0.003	1.005	1.00–1.01
Cholesterol (mg%)	185.30 ± 47.06	186.35 ± 47.64	180.6^ns^ ± 44.50	0.343	0.998	0.99–1.00	–	–	–
Sodium (mmol/l)	138.90 ± 4.35	139.01 ± 3.95	138.29^ns^ ± 5.67	0.140	0.963	0.91–1.01	–	–	–
LVEDV (ml)	88.43 ± 28.53	87.48 ± 26.44	92.93^ns^ ± 36.95	0.164	1.007	1.00–1.02	–	–	–
LVEF (%)	49.47 ± 7.73	49.48 ± 7.87	49.43^ns^ ± 7.13	0.913	0.998	0.96–1.04	–	–	–
BB	253 (56.22%)	213 (59.33%)	40^*^ (43.96%)	0.005	0.550	0.36–0.83	0.500	0.838	0.50–1.40
ACE-I	227 (50.44%)	189 (52.65%)	38^ns^ (41.76%)	0.051	0.660	0.44–1.00	0.524	0.840	0.49–1.44
ARB	29 (6.44%)	26 (7.24%)	3^ns^ (3.30%)	0.182	0.456	0.14–1.45	–	–	–
Statins	174 (38.67%)	155 (43.18%)	19^***^ (20.88%)	0.000	0.371	0.22–0.62	–	–	–
TD	80 (17.78%)	69 (19.22%)	11^ns^ (12.09%)	0.094	0.583	0.31–1.10	0.643	0.846	0.42–1.71
TLD	76 (16.89%)	55 (15.32%)	21^ns^ (23.08%)	0.070	1.571	0.96–2.56	0.059	1.750	0.98–3.13
ASS	210 (46.67%)	177 (49.30%)	33^*^ (36.26%)	0.020	0.601	0.39–0.92	0.143	0.675	0.40–1.14
AVK	63 (14.00%)	57 (15.88%)	6^***^ (6.59%)	0.027	0.391	0.17–0.90	0.017	0.330	0.13–0.82
Digoxin	41 (9.11%)	29 (8.08%)	12^ns^ (13.19%)	0.124	1.611	0.88–2.96	–	–	–
AA	74 (16.44%)	60 (16.71%)	14^ns^ (15.38%)	0.747	0.911	0.51–1.61	–	–	–
Oral anti-DM drugs	52 (11.56%)	42 (11.70%)	10^ns^ (10.99%)	0.840	0.934	0.48–1.80	–	–	–
Insulin therapy	27 (6.00%)	15 (4.18%)	12^**^ (13.19%)	0.000	3.030	1.65–5.57	0.003	2.832	1.44–5.58

^*^ *p* < 0.05, ^**^ *p* < 0.01, ^***^ *p* < 0.001

*ns* Not significant, *DDD* dual-chamber pacemaker, *VVI* single-chamber pacemaker, *VVI-ARVLL* single-chamber pacemaker with apical right ventricular lead location, *DDD-ARVLL* dual-chamber pacemaker with apical right ventricular lead location, *n-ARVLL* non-apical right ventricular lead location, *SSS* sick sinus syndrome, *A-V* atrial-ventricular block (degree II i III), *FA* atrial fibrillation, *CAD* coronary artery disease, *HA* arterial hypertension, *DM-2* type 2 diabetes, *LVEDV* left ventricular end-diastolic volume, *LVEF* left ventricular ejection fraction, *ACE-1* angiotensin-converting enzyme inhibitor, *ARB* angiotensin receptor blocker, *TD* thiazide derivative diuretic, *TLD* loop diuretic, *ASS* acetylsalicylic acid, *AVK* oral anti-coagulant, *AA* aldosterone antagonist, *anti-DM* antidiabetic

**Table 2:** Baseline female patient data and survival analysis (uni- and multivariable Cox regression)

Female	Survival	Death	Univariable Cox regression analysis			Multivariable Cox regression analysis		
No. of patients	193 (86.55%)	30 (13.45%)	*p*	HR	95% CI	*p*	HR	95% CI
Mean age at implantation	69.36 ± 9.58	75.23^***^ ± 4.47	0.001	1.144	1.05–1.24	0.007	1.116	1.03–1.21
DDD	138 (71.50%)	15^*^ (50.00%)	0.018	0.421	0.21–0.86	0.252	0.637	0.29–1.38
VVI	55 (28.50%)	15^*^ (50.00%)	–	–	–	–	–	–
VVI-ARVLL	40 (20.73%)	11^ns^ (36.67%)	–	–	–	–	–	–
DDD-ARVLL	99 (51.30%)	11^ns^ (36.67%)	–	–	–	–	–	–
n-ARVLL	54 (28.0%)	8^ns^ (26.7%)	0.938	0.968	0.43–2.18	–	–	–
SSS	104 (53.89%)	14^ns^ (46.67%)	0.458	0.762	0.37–1.56	–	–	–
A-V block	61 (31.61%)	13^ns^ (43.33%)	0.202	1.600	0.78–3.29	–	–	–
FA with bradycardia	28 (14.51%)	3^ns^ (10.00%)	0.511	0.671	0.20–2.21	–	–	–
CAD	48 (31.4%)	8^ns ^(26.7%)	0.886	1.061	0.47–2.38	–	–	–
HA	96 (49.74%)	12^ns^ (40.00%)	0.290	0.674	0.32–1.40	–	–	–
DM-2	36 (18.65%)	10^ns^ (33.33%)	0.062	2.063	0.97–4.41	0.070	2.380	0.93–6.07
Stroke	11 (5.70%)	3^ns^ (10.00%)	0.377	1.712	0.52–5.65	–	–	–
Hemoglobin (g/dl)	12.82 ± 1.44	12.27^*^ ± 1.71	0.045	0.811	0.66–0.99	0.255	0.867	0.68–1.11
Creatinine (µmol/l)	78.54 ± 25.06	108.49^*^ ± 85.24	0.001	1.007	1.00–1.01	0.208	1.003	1.00–1.01
Cholesterol (mg%)	192.38 ± 47.87	179.0^ns^ ± 46.63	0.174	0.994	0.98–1.00	–	–	–
Sodium (mmol/l)	139.37 ± 4.11	137.39^ns^ ± 7.39	0.022	0.917	0.85–0.99	0.047	0.926	0.86–1.00
LVEDV (ml)	79.65 ± 21.71	84.85^ns^ ± 34.04	0.358	1.010	0.99–1.03	–	–	–
LVEF (%)	50.21 ± 7.04	48.79^ns^ ± 7.74	0.426	0.970	0.90–1.04	–	–	–
BB	110 (56.99%)	15^ns^ (50.00%)	0.411	0.740	0.36–1.52	–	–	–
ACE-I	88 (45.60%)	9^ns^ (30.00%)	0.106	0.524	0.24–1.15	–	–	–
ARB	20 (10.36%)	1^ns^ (3.33%)	0.259	0.315	0.04–2.34	–	–	–
Statins	82 (42.49%)	4^**^ (13.33%)	0.005	0.220	0.08–0.63	0.019	0.260	0.08–0.80
TLD	43 (22.28%)	2^ns^ (6.67%)	0.067	0.260	0.06–1.10	0.126	0.318	0.07–1.38
TD	34 (17.62%)	9^ns ^ (30.00%)	0.118	1.866	0.85–4.08	–	–	–
ASS	84 (43.52%)	9^ns ^ (30.00%)	0.155	0.567	0.26–1.24	–	–	–
AVK	38 (19.69%)	1^ns^ (3.33%)	0.063	0.148	0.02–1.11	0.036	0.115	0.02–0.87
Digoxin	20 (10.36%)	6^ns^ (20.00%)	0.127	2.009	0.82–4.92	–	–	–
AA	32 (16.58%)	5^ns^ (16.67%)	0.998	0.999	0.38–2.61	–	–	–
Oral anti-DM drugs	16 (8.29%)	2^ns^ (6.67%)	0.803	0.833	0.20–3.50	–	–	–
Insulin therapy	8 (4.15%)	5^*^ (16.67%)	0.005	3.966	1.52–10.38	0.053	3.277	0.99–10.90

*p* < 0.05, ^**^ *p* < 0.01, ^***^ *p* < 0.001

*ns* Nonsignificant, *DDD* dual-chamber pacemaker, *VVI* single-chamber pacemaker, *VVI-ARVLL* single-chamber pacemaker with apical right ventricular lead location, *DDD-ARVLL* dual-chamber pacemaker with apical right ventricular lead location, *n-ARVLL* non-apical right ventricular lead location, *SSS* sick sinus syndrome, *A-V* atrial-ventricular block (degree II i III), *FA* atrial fibrillation, *CAD* coronary artery disease, *HA* arterial hypertension, *DM-2* type 2 diabetes, *LVEDV* left ventricular end-diastolic volume, *LVEF* left ventricular ejection fraction, *ACE-1* angiotensin-converting enzyme inhibitor, *ARB* angiotensin receptor blocker, *TD* thiazide derivative diuretic, *TLD* loop diuretic, *ASS* acetylsalicylic acid, *AVK* oral anti-coagulant, *AA* aldosterone antagonist, *anti-DM* antidiabetic

**Table 3:** Baseline male patient data and survival analysis (uni- and multivariable Cox regression)

Male	Survival	Death	Univariable Cox regression analysis			Multivariable Cox regression analysis		
No. of patients	166 (73.13%)	61 (26.87%)	*p*	HR	95% CI	*p*	HR	95% CI
Mean age at implantation	67.05 ± 10.48	71.29^**^ ± 7.31	0.005	1.051	1.02–1.09	0.160	1.023	0.99–1.06
DDD	118 (71.08%)	31^**^ (50.82%)	0.002	0.459	0.28–0.76	0.080	0.620	0.36–1.06
VVI	48 (28.92%)	30^**^ (49.18%)	–	–	–	–	–	–
VVI-ARVLL	26 (15.66%)	23^***^ (37.70%)	–	–	–	–	–	–
DDD-ARVLL	67 (40.36%)	19^ns ^(31.15%)	–	–	–	–	–	–
n-ARVLL	73 (46.4%)	19^ns ^(31.1%)	0.051	0.582	0.34–1.00	0.042	0.555	0.31–0.98
SSS	57 (34.34%)	22^ns ^(36.07%)	0.815	1.064	0.63–1.80	–	–	–
A-V block	87 (52.41%)	32^*^ (52.46%)	0.972	1.009	0.61–1.67	–	–	–
FA with bradycardia	22 (13.25%)	7^ns ^(11.48%)	0.703	0.858	0.39–1.89	–	–	–
CAD	67 (40.3%)	18^ns ^(18.3%)	0.119	0.645	0.37–1.12	–	–	–
HA	83 (50.00%)	22^ns ^(36.07%)	0.064	0.610	0.36–1.03	0.352	0.745	0.40–1.38
DM-2	38 (22.89%)	14^ns ^(22.95%)	0.951	1.019	0.56–1.85	–	–	–
Stroke	6 (3.61%)	4^ns ^(6.56%)	0.395	1.553	0.56–4.28	–	–	–
Hemoglobin (g/dl)	13.88 ± 1.42	13.59^ns^ ± 2.08	0.180	0.894	0.76–1.05	–	–	–
Creatinine (µmol/l)	89.90 ± 20.94	111.5^***^ ± 39.17	0.000	1.018	1.01–1.02	0.001	1.012	1.01–1.02
Cholesterol (mg%)	179.4 ± 46.59	181.5^ns^ ± 43.78	0.837	1.001	0.99–1.01	–	–	–
Sodium (mmol/l)	138.6 ± 3.71	138.8^ns^ ± 4.54	0.852	1.006	0.94–1.08	–	–	–
LVEDV (ml)	95.39 ± 28.45	96.12^ns^ ± 38.06	0.358	1.010	0.99–1.03	–	–	–
LVEF (%)	48.69 ± 8.52	49.72^ns^ ± 6.95	0.426	0.970	0.90–1.04	–	–	–
BB	103 (62.05%)	25^**^ (40.98%)	0.003	0.463	0.28–0.77	0.147	0.639	0.35–1.17
ACE-I	101 (60.84%)	29^ns ^(47.54%)	0.058	0.615	0.37–1.02	0.775	0.911	0.48–1.73
ARB	6 (3.61%)	2^ns ^(3.28%)	0.823	0.851	0.21–3.48	–	–	–
Statins	73 (43.98%)	15^*^ (24.59%)	0.007	0.450	0.25–0.81	0.611	0.836	0.42–1.67
TLD	26 (15.66%)	9^ns ^(14.75%)	0.717	0.877	0.43–1.78	–	–	–
TD	21 (12.65%)	12^ns ^(19.67%)	0.142	1.605	0.85–3.02	–	–	–
ASS	93 (56.02%)	24^*^ (39.34%)	0.018	0.539	0.32–0.90	0.425	0.781	0.43–1.43
AVK	19 (11.45%)	5^ns ^(8.20%)	0.402	0.676	0.27–1.69	–	–	–
Digoxin	9 (5.42%)	6^ns ^(9.84%)	0.200	1.736	0.75–4.03	–	–	–
AA	28 (16.87%)	9^ns ^(14.75%)	0.718	0.878	0.43–1.78	–	–	–
Oral anti-DM drugs	26 (15.66%)	8^ns ^(13.11%)	0.588	0.814	0.39–1.71	–	–	–
Insulin therapy	7 (4.22%)	7^ns ^(11.48%)	0.017	2.609	1.19–5.74	0.124	2.024	0.82–4.97

^*^ *p* < 0.05, ^**^ *p* < 0.01, ^***^ *p* < 0.001

*ns* nonsignificant, *DDD* dual-chamber pacemaker, *VVI* single-chamber pacemaker, *VVI-ARVLL* single-chamber pacemaker with apical right ventricular lead location, *DDD-ARVLL* dual-chamber pacemaker with apical right ventricular lead location, *n-ARVLL* non-apical right ventricular lead location, *SSS* sick sinus syndrome, *A-V* atrial-ventricular block (degree II i III), *FA* atrial fibrillation, *CAD* coronary artery disease, *HA* arterial hypertension, *DM-2* type 2 diabetes, *LVEDV* left ventricular end-diastolic volume, *LVEF* left ventricular ejection fraction, *ACE-1* angiotensin-converting enzyme inhibitor, *ARB* angiotensin receptor blocker, *TD* thiazide derivative diuretic, *TLD* loop diuretic, *ASS* acetylsalicylic acid, *AVK* oral anti-coagulant, *AA* aldosterone antagonist, *anti-DM* antidiabetic

n-ARVLL was more frequent in women with sick sinus syndrome. The female subgroups selected on the basis of lead location did not differ significantly in the type of implanted stimulator, frequency of atrioventricular block, atrial fibrillation with low ventricular action, coronary artery disease, arterial hypertension, type 2 diabetes, and brain stroke in their medical history.

In the male subgroup, n‑ARVLL was significantly dominant in the group of patients with atrioventricular block and in patients with a history of brain stroke. No statistically significant differences were observed in the remaining parameters.

Women were older than men, they suffered from coronary disease less frequently, and significantly more often had the RV lead in the apical location. Women had a pacemaker implanted more often because of sick sinus syndrome and less often because of atrioventricular conduction disorders (Table 4).

**Table 4:** Study group characteristics according to sex and RV lead location

	F *n* = 223			M *n* = 227			F vs. M
	ARVLL *n* = 161	n-ARVLL *n* = 62		ARVLL *n* = 135	n-ARVLL *n* = 92		*p* < 0.01
Mean age at implantation	70.03 ± 9.32	70.47 ± 9.23	ns	68.94 ± 10.14	67.10 ± 9.48	ns	*p* < 0.01
DDD	110 (68.3%)	43 (69.4%)	ns	86 (63.7%)	63 (68.5%)	ns	ns
SSS	76 (47.2%)	42 (67.7%)	*p* < 0.05	42 (31.1%)	37 (40.2%)	ns	*p* < 0.001
A-V block	62 (38.5%)	12 (19.4%)	ns	79 (58.5%)	40 (43.5%)	*p* < 0.05	*p* < 0.001
FA with bradycardia	23 (14.3%)	8 (12.9%)	ns	14 (10.4)	15 (16.3%)	ns	ns
CAD	42 (26.1%)	14 (22.6%)	ns	51 (37.8%)	34 (37.0%)	ns	*p* < 0.01
HA	78 (48.4%)	30 (48.4%)	ns	66 (48.9%)	39 (42.4%)	ns	ns
DM-2	31 (19.3%)	15 (24.2%)	ns	33 (24.4%)	19 (20.7%)	ns	ns
Stroke	9 (5.56%)	5 (7.94%)	ns	3 (2.22%)	7 (7.61%)	*p* < 0.05	ns

*p* < 0.05, ^**^ *p* < 0.01, ^***^ *p* < 0.001

*ARVLL* apical right ventricular lead location, *n-ARVLL* non-apical right ventricular lead location, *ns* nonsignificant, *DDD* dual-chamber pacemaker, *SSS* sick sinus syndrome, *A-V block* atrioventricular block (degree II i III), *FA* atrial fibrillation, *CAD* coronary artery disease, *HA* arterial hypertension, *DM-2* type 2 diabetes, *F* Female, *M* Male

### Univariable Cox regression

In the entire group of patients, univariable Cox hazard analysis indicated that the following factors were of positive prognostic significance: female sex (HR = 0.463); DDD pacemaker implantation (HR = 0.441); concurrent arterial hypertension (HR = 0.623); BB application (HR = 0.550); statins (HR = 0.371); ASS (HR = 0.601); ACE-I (HR = 0.660); thiazide-derivative diuretic: (HR = 0.583); and AVK (HR = 0.391).

The increased risk of death in the entire group potentially concerned the patients of more advanced age (HR = 1.063), with higher creatinine concentrations (HR = 1.008), requiring loop diuretics (HR = 1.571), and requiring insulin therapy (HR = 3.030).

In the female subgroup, the following factors had potentially protective significance: DDD pacemaker implantation (HR = 0.421); thiazide-derivative diuretics (HR = 0.260); statins (HR = 0.220); and AVK (HR = 0.148) as well as higher hemoglobin concentrations (HR = 0.811) and lower sodium concentrations (HR = 0.917). A potential indicator of worse prognosis was age (HR = 1.144), creatinine concentration (HR = 1.007), and diabetes (HR = 2.063) especially diabetes requiring insulin therapy (HR = 3.966).

In the male subgroup, the following factors were of potentially advantageous importance: DDD pacemaker implantation (HR = 0.459); n‑ARVLL (HR = 0.582); arterial hypertension (HR = 0.610); BB application (HR = 0.463); ACE-I (HR = 0.615); statins (HR = 0.450); and ASS (HR = 0.539). Age was also a potential indicator of worse prognosis (HR = 1.051), as was creatinine concentration (HR = 1.018) and diabetes requiring insulin therapy (HR = 2.609).

### Multivariable Cox regression

In the entire patient group, multivariable Cox regression showed that the following factors were of positive prognostic significance: female sex (HR = 0.426); DDD pacemaker implantation (HR = 0.526); and the application of oral anti-coagulants (HR = 0.330). Age (HR = 1.047), insulin therapy (HR = 2.832), and higher creatinine concentration (HR = 1.005) had a statistically significant influence on the risk of early death in the entire patient group.

Sodium concentration had positive prognostic significance in the female subgroup (HR = 0.926), as did oral anticoagulant (HR = 0.115) and statin use (HR = 0.260). An adverse prognostic factor was age (HR = 1.116).

In the male subgroup, a protective influence of n‑ARVLL was noted (HR = 0.555), with a worse prognosis seen in patients with elevated creatinine levels (HR = 1.012).

### Kaplan–Meier survival curves and log-rank analysis

In the entire patient group, despite the distribution of the survival curves, log-rank analysis did not indicate a statistically significant influence of RVLT location on survival in the 4‑year observation period (*p* = 0.287).

**Fig. 1:**
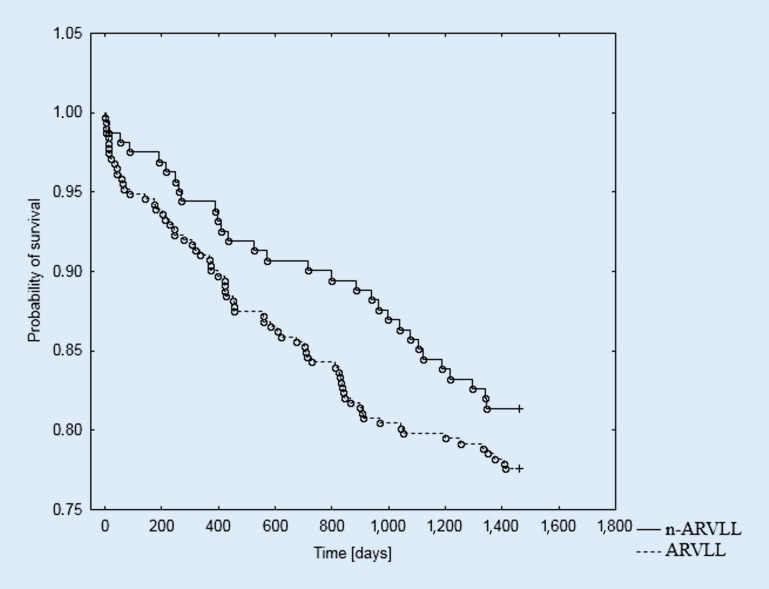
Probability of survival in the study group depending on the location of the right ventricular lead; log-rank *p* = 0.287. *ARVLL* apical right ventricular lead location, *n-ARVLL* non-apical right ventricular lead location

**Fig. 2:**
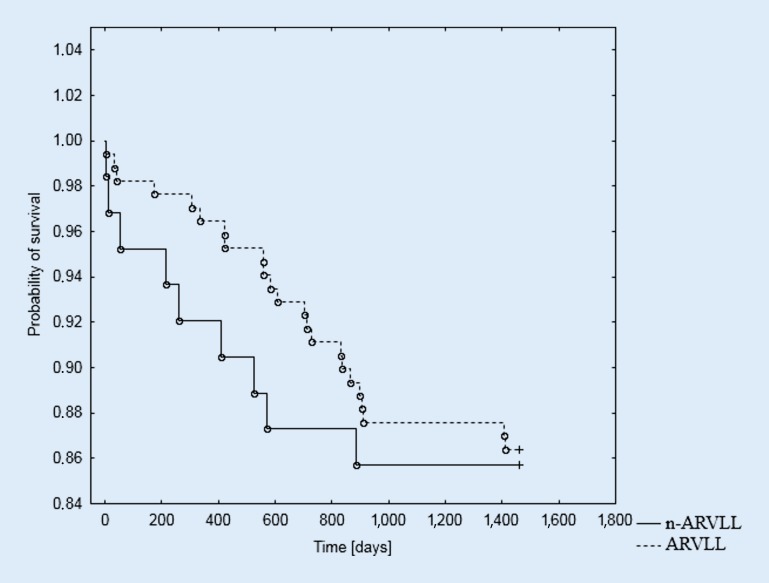
Probability of survival in the female subgroup depending on the location of the right ventricular lead; log-rank *p* = 0.823. *ARVLL* apical right ventricular lead location, *n-ARVLL* non-apical right ventricular lead location

**Fig. 3:**
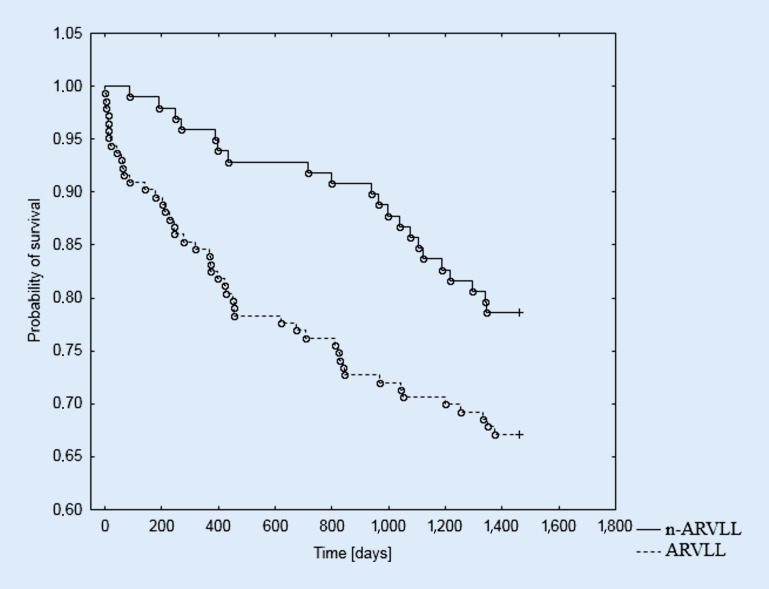
Probability of survival in the male subgroup depending on the location of the right ventricular lead; log-rank *p* = 0.028. *ARVLL* apical right ventricular lead location, *n-ARVLL* non-apical right ventricular lead location

The log-rank analysis indicated a statistically significant influence of the RVLT location in the distribution of the curves, showing the survival time during the 4‑year observation in the male subgroup (*p* = 0.028). However, no prognostic significance was found for RVLT location in the female subgroup (*p* = 0.823). The Kaplan–Meier survival curves for the female and male groups according to RVLT location are presented in Figs. 1, 2 and 3.

## Discussion

In this case-control study, we found that non-apical pacing has a positive influence on the survival rate, with statistically significant differences seen in the male subgroup.

The number of studies was smaller in comparison with large clinical studies examining the influence of electrotherapy on the cardiovascular system [29, 29–31]. The patients analyzed here were younger than the population evaluated in other studies [28, 29]. However, the indications for pacemaker implantation and the observation period were similar to those in other studies [29, 31, 32]. In the present study group, a significant percentage of patients had dual-chamber pacemaker implantation (67.11%), which differentiates it from previous papers, especially those with a longer observation period [28, 30].

The mortality rate during the 4‑year observation period was similar to the rate reported for the study by Udo et al., in which the survival rate after 3 and 5 years was 81% and 69%, respectively [31], and lower than the one reported by Shiomo et al., where 67.2% of patients survived in the 4‑year observation period [32]. The population evaluated by Pyatt et al. was almost two times larger and approximately 8 years older than the one in our study; the men constituted 51.9% of the research group, there were more patients with atrioventricular conduction disorders, and thus pacing in DDD mode was dominant. Fewer patients suffered from ischemic heart disease, diabetes, or past neurological incidents. Older age at the time of pacemaker implantation, cardiomyopathy in medical history, valve defects, VVI pacing, as well as male sex were among the factors with negative prognostic influence [29]. In the study by Udo et al., the indicators of total mortality rate were older age, male sex, BMI, concurrent ischemic heart disease, circulatory insufficiency, and atrioventricular conduction disorders [31].

In the analysis conducted by Brunner et al., concerning the survival of 6505 patients during an 8-year observation period, the negative influence of the following parameters on the survival rate was reported: older age at the time of device implantation, male sex, earlier period of pacemaker implantation, pacing in VVI mode, concurrent atrial fibrillation, fainting or total lack of fainting accompanying bradycardia, and lack of pre-MAS symptoms prior to stimulator implantation [28].

As in other studies, in our entire patient group, female sex (despite older age among women) [28, 29, 31], dual-chamber pacing, and anti-coagulant AVK therapy [33, 34] had prognostic significance, whereas the indicators of poor prognosis were higher creatinine concentration [35], loop diuretic application, and diabetes requiring insulin therapy [36]. Although the univariable analysis indicated a positive prognostic influence of BB, ACE-I, and statin application, this was not reflected in the multivariable model. However, in the female group, the application of statins and AVK improved the prognosis, and the following factors had negative prognostic significance: age and diabetes requiring insulin therapy as well as lower sodium concentration. In the male subgroup, the non-apical location of the RV lead had a positive influence on survival, whereas high creatinine concentration was a negative factor.

In contrast to other studies, we did not find any influence of coronary artery disease, arterial hypertension, and atrial fibrillation on the long-term prognosis of our study group [28, 31]. This can be explained by the fact that individuals with stable cardiovascular system disorders, receiving optimal pharmacotherapy, were included in the research. In addition, the patients included in the study had left ventricular contraction function.

Studies comparing single- and dual-chamber pacing indicate that physiological pacing is associated with an improved life quality and physical efficiency as well as with a lower frequency of atrial fibrillation episodes [16, 18, 28, 37], brain strokes, and heart insufficiency [12, 16].

The positive aspects of this type of pacing can be hampered by the unnecessarily high frequency of pacing [16, 17] or ventricular contraction desynchronization connected with the apical localization of the ventricular lead. The partially different research results [16, 18, 28, 38] on the prognosis can be due to the varied percentages of ventricular pacing, which, along with its increase, led both to an increase in hospitalization risk due to heart failure [16, 17] and in a lower survival rate [17].

In the present study group, almost 70% of patients had a dual-chamber pacemaker implanted and the percentage of patients with the implanted device working in VVI mode was low along with a maintained sinus rhythm. Owing to the retrospective nature of the study, we do not have data available on the percentage of ventricular pacing; however, the significant majority of implanted dual-chamber pacing devices had an active self-organizing atrioventricular search algorithm, which is especially important in patients with sick sinus syndrome [16, 18, 39–41].

The multivariable analysis conducted in the entire patient group did not show a significant prognostic significance for RVLT location. Its non-apical location was the only parameter with positive prognostic significance in the male subgroup. The literature on the prognostic importance of the RV pacing site is not extensive and does not provide consistent results. In our opinion, the positive prognostic effect of non-apical pacing in the male subgroup was associated with sex, which can be the result of the more frequent occurrence of atrioventricular conduction disorders requiring a higher percentage of pacing as compared with the female subgroup in which sick sinus syndrome was dominant.

Dąbrowska Kugacka et al., conducting a study of 122 patients aged 69 ± 11 years, with typical indications for pacemaker implantation randomized to subgroups with apical vs. non-apical RV pacing, did not report a statistically significant reduction in general mortality rate as well as death due to cardiovascular reasons in patients without RVA vs. RVA pacing. In the multivariable analysis, male sex and older age at the time of pacemaker implantation were among factors contributing to a worse prognosis of general mortality rate [42]. Similarly, in the study conducted by Kypty et al. on 98 patients with atrioventricular block including 53 patients with n‑ARVLL, a total of nine patients died in the course of an 18-month observation period, among whom were five in the RVNA subgroup, reaching a statically nonsignificant difference in the survival analysis.

In turn, a positive prognostic effect of RVOT (right ventricle outflow tract) pacing was reported in the study by Vanerio et al. The authors stated that in a group of 150 patients with implanted pacemakers, 18 patients with RVOT pacing and 49 patients with apical pacing died during a middle- and long-term observation period, obtaining a statistically significant result in the log-rank analysis (*p* = 0.02) [43].

Despite the restrictions, the results we have obtained are complementary to current research and confirm the positive influence of non-apical pacing on long-term prognosis. In order to make the aforementioned observations objective, it is necessary to conduct further randomized prospective studies in this field.

### Study limitations

This study’s limitations include the retrospective nature of the research and the lack of randomization as well as data on the percentage of RV pacing. The heterogeneity of the evaluated population in terms of primary diseases is a further limitation.

### Conclusion

The non-apical location of the RVLT has a positive influence on patient prognosis, with statistical significance found for the male subgroup in this study. However, these observations require verification in further randomized studies.
